# Clinical outcomes of posterior spinal stabilization with rigid vertical strut and spinal process wires (the Adeolu’s technique) in a developing country

**DOI:** 10.11604/pamj.2017.26.84.8278

**Published:** 2017-02-21

**Authors:** Taopheeq Bamidele Rabiu

**Affiliations:** 1 Division of Neurological Surgery, Department of Surgery, LAUTECH Teaching Hospital, Osogbo, Nigeria

**Keywords:** Spinal stabilization, vertical struts, spinal process wires, Adeolu´s technique, Nigeria, developing country

## Abstract

**Introduction:**

Conventional instrumentation for spinal stabilization is beyond the reach of many patients in developing countries. A low-cost and easily-available method of spinal stabilization using vertical struts and spinal process wires (Adeolu's technique) was recently introduced in Nigeria. We describe the clinical outcomes of a prospective series of patients managed using the technique.

**Methods:**

From 2011 to 2012, we performed posterior spinal stabilization in eighteen patients using the technique. Primary outcomes were radiological evidence of rigid stabilization and mobilization without restrictions referable to the procedure in the immediate post-operative period. Implant rotation, migration, back-out, fracture, wound infection, worsening neurological status and need for implant removal were secondary measures. Overall patient satisfaction was assessed using a five-point Likert scale. The average follow-up period was 11.6 months.

**Results:**

The average age of the patients was 45.8 years. Trauma with unstable spinal fractures (11), spondylosis (5), and thoracic extra-dural tumour (2) were the indications for surgery. The average number of spinal levels stabilized was 6. All patients had satisfactory primary outcomes. Implant rotation occurred in 3 patients (16.7%). There was no case of implant migration, back-out or fracture. Superficial surgical site infection occurred in one patient. There was no need to remove the implant in any subject and none had post-operative worsening of neurological status. The overall patient satisfaction was good with 17 patients (94.4%) reporting “highly satisfied” or “satisfied” with the surgical procedure.

**Conclusion:**

The technique offers utility in a wide range of spinal pathologies and short-term clinical outcomes are good.

## Introduction

Since the first description of spinal instrumentation in the treatment of Pott's disease, several techniques, methods and materials have been used in spinal stabilization or fusion [1, 2]. Examples of instrumentations in current use for spinal stabilization are pedicle screws (with rods or plates), translaminar or facet screws and Hartshill rectangle [2]. In an attempt to reduce adjacent segment diseases through the alteration of load bearing and control of abnormal motion, many dynamic stabilization devices such as Dynesys (Dynamic Neutralization System for the Spine) have also been introduced [2]. Many of these techniques have limited use in developing countries not only as a result of poor economies which make the implants out of reach of the vast majority of patients but also because intra-operative imaging facilities which are needed for their safe use are unavailable in many hospitals in the developing world. In addition, it is imperative to note that attempts at transferring tools and equipments for use in many developing countries may not always be successful as some of them may not be practical due to prohibitive maintenance costs and damage by the environment [3]. Against this gloomy background, locally adaptable technologies are being introduced for the use of neurosurgeons working in resource-constrained environments [4]. In Nigeria, a low-cost and easily available method of spinal stabilization using rigid vertical struts and spinal process wires was recently introduced by Adeolu et al [5]. Praising the technique, the authors stated that it “encompasses simplicity and low cost comparable to sublaminar wiring and vertical strut but without the complications” [5]. This is in reference to its similarity with spinal fusion using sublaminar wires and vertical struts which were abandoned as a result of complications such as spinal cord injury and spinal canal compromise [6]. In this paper, we describe the clinical outcomes of a prospective series of Nigerian patients with various spinal pathologies managed using the technique.

## Methods

***Study design and patient demographics:*** Eighteen patients who had operative spinal stabilization using the Adeolu's technique performed by the author between January 2011 and November 2012 at three Nigerian hospitals: Federal Medical Centre, Ido-Ekiti, Onward Specialist Hospital, Osogbo and LAUTECH Teaching Hospital, Osogbo, are included in the study. A prospective serial database of the patient was kept. The database included the patient demographics, nature of clinical diagnoses, details of operations including the procedures performed and findings, and clinical outcomes. The patients have been followed up for 2 to 23 months (Average: 11.6 months).

**Figure 1:**
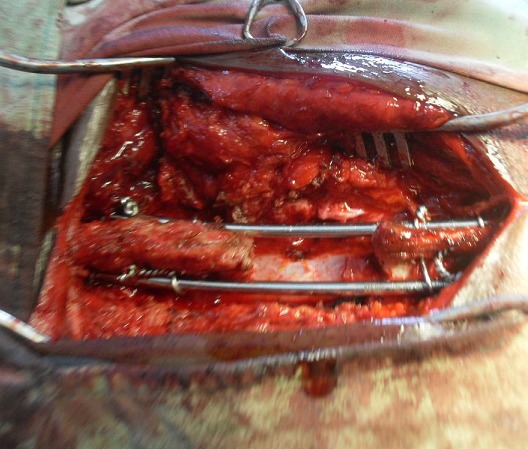
Intra-operative view of the implants just before wound closure

***Operative procedures:*** The technique proper is as detailed by Adeolu et al [5]. Patients with traumatic spinal subluxation had pre-operative complete or partial closed postural reductions. All the procedures were performed under general anaesthesia with the patient in mostly prone position. Park-bench position was used in 2 cases involving obese patients. Standard midline approach to the spine is employed. Laminectomy is done as necessary. Holes are made at the bases of the spinous processes of the vertebrae to be instrumented (usually two levels above and below the site{s} of instability) and loops of spinal wires passed in reversed order through them. Rush nails (bent at right angle at the end) are passed through the loops of wires (one on each side). The wire loops are then twisted and tightened around the nails for a rigid spinal construct. We made use of size 3.5-4mm Rush nails depending on the body build of the patient. We routinely used size 20G spinal wires. Bone grafts are placed beside the rods as necessary. We did not use drains. The implants were inserted under direct vision without magnification or use of intra-operative imaging. An example of the intra-operative view of the implants just before wound closure is shown in Figure 1.

**Figure 2:**
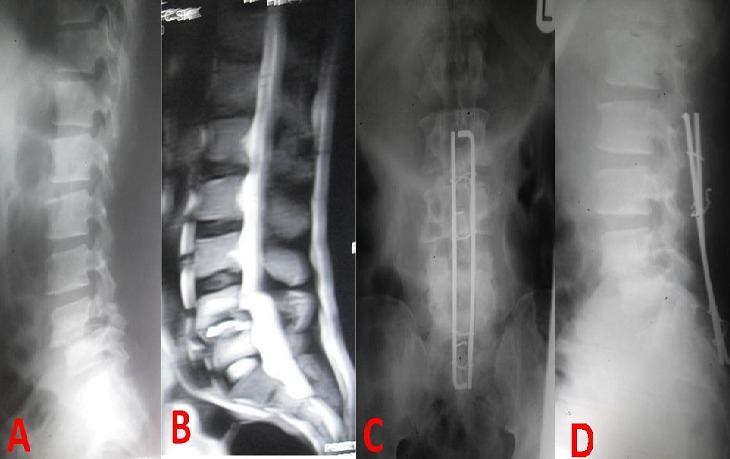
Pre-operative (A,B) and post-op (C, D) images in a patient with grade II L4 spondylolisthesis

**Figure 3:**
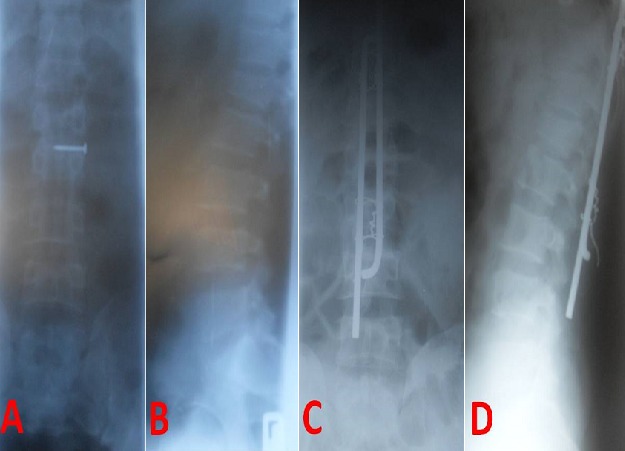
Pre- (A, B) and post-operative (C, D) X-rays in a trauma patient with L1 compression fracture. The nail over L1 spinous process (A) was used for pre-operative spinal marking. D shows slight rotation of one vertical strut.

***Radiological studies:*** All the patients underwent pre-operative X-rays. Except for some of the trauma patients, Magnetic Resonance imaging (MRI) was also performed for diagnoses and planning of surgical approaches. The length of the implants is determined on the pre-operative imaging. In the immediate post-operative period, usually when the patient is being transferred from the operation theatre to the ward or within 48 hours of surgery, anterio-posterior and lateral X-ray views of the instrumented region are obtained to ascertain the correctness of levels of surgery and position of the implants. Spinal X-rays are obtained further in the course of the follow-up to evaluate the integrity of the spine and the implants in some of the patients and in a few patients, in the event of unexpected new-onset pain. Figure 2 and Figure 3 give examples of pre-operative and post-operative imaging findings in the study.

***Primary outcomes:*** The primary outcomes were radiological evidence of rigid spinal stabilization and ability to mobilize without restrictions referable to the procedure in the immediate post-operative period.

***Secondary outcomes:*** Implant rotation, migration, back-out and fracture on post-operative X-rays as well as wound infection, new-onset pain, worsening of neurological status and need for implant removal were secondary measures of outcome.

***Measure of satisfaction:*** Overall patient satisfaction with the procedure was assessed using a five-point Likert scale obtained at the last post-operative out-patient clinic visit or via telephone calls to the patients in a few cases. The patients were asked to select one of five fixed items viz: “highly satisfied”/“satisfied”/“neutral”/“unsatisfied”/“highly unsatisfied” to describe their overall level of satisfaction or otherwise with the stabilization procedure.

***Data analysis:*** Simple descriptive statistics have been performed.

## Results

***Patient demographics:*** The patients and their specific pathologies and details of operations are listed in Table 1. The average age of the patients was 45.8 years (range: 30-70 years). Trauma with unstable spinal fractures with or without mal-alignment and cord compression (11), spondylosis (5) including 3 cases of spondylolisthesis, and thoracic extra-dural tumour (2) were the indications for surgery. The average number of spinal levels stabilized was 6 (range: 5-10).

**Table 1:** Patients’ characteristics, surgical diagnoses and procedures

S/N	Age (Years)	Sex	Indication for Surgery	Operation Performed	No of Spinal Levels stabilized
1	40	M	Trauma with L1 burst fracture and cord compression	Decompressive L1 laminectomy + T11-L3 spinal stabilization	5
2	41	F	Traumatic L1 paraparesis + L5/S1 ant. Subluxation & L5 laminar fracture	L5 laminectomy + L3 – S2 spinal stabilization	5
3	30	M	Traumatic T8 paraplegia with T8+T9 laminar Fractures	T8 and T9 laminectomies + T6-T11 spinal stabilization	6
4	32	M	Trauma tic T4 paraplegia with T4/5 ant. Subluxation + T4 & 5 laminar fracture	T4 and T5laminectomies + T2 – T7 spinal stabilization	6
5	56	M	Traumatic T10 paraplegia with T10/11 ant. Subluxation	T10 laminectomy + T8- T12 spinal stabilization	5
6	42	M	Traumatic L1 paraparesis with L1/2 ant. Subluxation + L2 compression fracture	Decompressive L1 laminectomy + T11 – L3 spinal stabilization	5
7	52	F	T8 myelopathy 2°Extradural spinal metastasis	T6 – T9 laminectomies + tumour excision + T4 – T11 spinal stabilization	8
8	47	M	Lumbar spondylosis + canal stenosis	Decompressive L1,2,4,5 laminectomies+multileveldiscectomies & foraminotomies + T10 – S2 spinal stabilization	10
9	40	M	Traumatic T10 paraplegia	T10+11 laminectomies + T8 – L1 spinal stabilization	6
10	70	F	L4 spondylolisthesis+ L4/5 & L5/S1 disc protrusions	L4 +5 laminectomies + L4/5 & L5/S1 discectomies + L2 – S2 spinal stabilization	6
11	70	F	L2 paraparesis 2°Extradural spinal tumour	L1 – 3 laminectomies + tumour excision + T11 – L5 spinal stabilization	7
12	31	M	Traumatic T12 myelopathy + L2 vertebral fracture	T12 – L4 spinal stabilization	5
13	50	M	L4 spondylolisthesis+ canal stenosis	L4 Gill’s procedure + L2 – S1 spinal stabilization	5
14	35	M	Traumatic T9 paraplegia	T7 laminectomy + T5 – T9 spinal stabilization	5
15	43	M	L4 spondylolisthesis+ canal stenosis	Decompressive L4 laminectomy + Bilateral L4/5 foraminotomies + L2 – S1 spinal stabilization	6
16	40	M	Traumatic L2 paraplegia	L2 laminectomy + T12 – L4 spinal stabilization	5
17	60	F	Severe Lumbar spondylosis + long segment canal stenosis	L2 – 5 laminectomies + Bilateral L2/3 – L5/S1 foraminotomies + T12 – S2 spinal stabilization	8
18	31	F	Traumatic T12 paraplegia	T12 + L1 laminectomies + T10 – L3 spinal stabilization	6

***Outcomes:*** All patients had satisfactory primary outcomes. Implant rotation occurred in 3 patients (16.7%). There was no recorded case of implant migration, back-out or fracture. Superficial surgical site infection occurred in one patient (Table 2). There was no need to remove the implant in any of the subjects and none of them had post-operative worsening of neurological status. The overall patient satisfaction was good with 17 patients (94.4%) reporting “highly satisfied” or “satisfied” with the surgical procedure (Table 3).

**Table 2:** Profile of secondary outcomes

Complication	No of patients	%
Implant rotation	3	16.7
Implant migration	0	0.0
Implant back-out	0	0.0
Implant fracture	0	0.0
Wound infection	1	0.6
Need for implant removal	0	0.0
Post-operative neurological deterioration	0	0.0

**Table 3:** Overall patient satisfaction measured with a Likert scale

Likert item	Number of patients (%)
Highly satisfied	13 (72.2)
Satisfied	4 (22.2)
Neutral	1 (5.6)
Unsatisfied	0 (0.0)
Highly unsatisfied	0 (0.0)

## Discussion

Developing appropriate technologies for neurosurgeons working in resource-constrained settings is a strategy through which the neurosurgical care of people in the developing world can be tremendously improved [4, 7]. Spinal instrumentation using cheap, simple and locally-made implants have been shown to have immediate and long term results comparable to prohibitively expensive and difficult to use imported ones [8]. It is in this context that technologies and techniques such as the one been evaluated in this study, developed for use in the developing countries need to be situated. Spinal stabilization in spinal cord injury (as occurred in a large proportion of the study subjects) has been shown to facilitate early post-operative mobilization, reduces complications of cord injury and results in reduced length and cost of hospital stay [9–11]. The goal of early mobilization was effectively met in the study subjects as shown by the 100% satisfactory primary outcome. The high level of overall patient satisfaction may reflect the fact that their early mobilization resulted in reduced length of stay in the hospital and a relative benefit of overall hospitalization cost-reduction. The observed implant rotation in 3 patients was thought to have occurred while the loops of spinal process wires were being tightened at the cranial and caudal ends. However, there was no clinical problem resulting from the rotation in any of the patients and as such there was no need for re-operation. After these observations, we have paid more attention to the positioning of the nails during wire tightening and prevented the complication in subsequent patients.

As observed by Adeolu et al [5], the technique has the inherent problem of not able to reduce or realign displaced vertebrae. For the patients with traumatic spinal mal-alignment, we tackled this problem through partial or complete postural reduction before the operative spinal stabilization. For the patients with spondylolisthesis, it has been shown that performing nerve root decompression without fusion, that is, Gill's procedure and also without instrumented realignment of the spine resulted in good long-term outcome [12]. Therefore, while achieving the results of Gill's procedure by decompressing the neural elements, our addition of spinal stabilization using the Adeolu's technique gives reassurance that the need for re-operation (which may not be financially feasible) in our patients, if any, would be very minimal. We have not provided the cost-benefit analysis of the technique compared with conventional spinal fusion instrumentations such as pedicle screws for obvious reason. Its low cost (about N5, 000.00 or $31.25) is definitely not comparable to the high costs of other implants which are beyond the reach of the average Nigerian patient. However, whether or not spinal stabilization using this technique offers an objective and superior cost-benefit compared with conservative care in our local spinal injury patients requires a randomized study. Also, tests of in-vivo biomechanical strengths of the implants were not conducted in our patients. A study involving serial X-rays of the spine under dynamic stresses may be needed to ascertain the ability of the implants to withstand biomechanical stresses. However, what is important ultimately, is the patient satisfaction and the fact that the technique affords satisfactory spinal stabilization, at least in the short term, as unlike arthroplasty, the surgery does not need to last as long as the implant because it is intended to maintain spinal stability pending bony fusion [8].

## Conclusion

We have provided the first evaluation of the Adeolu's technique in a cohort of neurosurgical patients in a developing country with resource constraints. The technique offers utility in a wide range of spinal pathologies and short-term clinical outcomes are good.

### What is known about this topic

- Several techniques, methods and materials are in use for spinal stabilization or fusion.
- Many of the available techniques have limited use in developing countries as a result of high cost and absence of intra-operative imaging facilities.
- Attempts at transferring tools and equipments for use in many developing countries may not always be successful due to prohibitive maintenance costs and damage by the environment.

### What this study adds

- Evaluation of a low-cost and easily available method of spinal stabilization using rigid vertical struts and spinal process wires.
- Good clinical outcomes of a prospective series of Nigerian patients with various spinal pathologies managed using the Adeolu's technique.
- Low complication rates with the use of the technique.
